# Why is there a gap in self-rated health among people with hypertension? A decomposition of determinants and rural–urban differences

**DOI:** 10.21203/rs.3.rs-3111338/v1

**Published:** 2023-06-30

**Authors:** Chris Mweemba, Wilbroad Mutale, Felix Masiye, Peter Hangoma

**Affiliations:** 1. Department of Health Policy and Management, School of Public Health, P.O. Box 50110, Ridgeway Campus, University of Zambia, Lusaka, Zambia; 2. Department of Economics, School of Humanities and Social Science, P.O Box 32379, Great East Road Campus, University of Zambia, Lusaka, Zambia; 3. Chr. Michelson Institute (CMI), Bergen, Norway; 4. Bergen Center for Ethics and Priority Setting in Health, University of Bergen, Bergen, Norway

**Keywords:** Self-rated health, hypertension, Blinder-Oaxaca decomposition, inequality, HIV/AIDS, Zambia, Household consumption expenditure

## Abstract

**Background:**

Hypertension affects over one billion people globally and is one of the leading causes of premature death. The low- and middle-income countries, especially the sub-Saharan Africa region, bear a disproportionately higher share of hypertension globally. Recent evidence shows a steady shift in the burden of hypertension from the more affluent and urban population towards the poorer and rural communities. Our study examined inequalities in self-rated health among people with hypertension and whether there is a rural-urban gap in the health of these patients. We then quantified factors driving the health gap. We also examined how much HIV accounts for differences in self-rated health among hypertension patients due to the relationship between HIV, hypertension and health in sub-Saharan Africa.

**Methods:**

We utilized the Zambia Household Health Expenditure and Utilization Survey for the data on SRH and other demographic and socioeconomic controls. District HIV prevalence information was from a previous study. The linear probability model provided a preliminary assessment of the association between self-rated health and independent variables. We then used the Blinder-Oaxaca decomposition to identify self-rated health inequality between urban and rural patients and determine determinants of the health gap between the two groups.

**Results:**

Advanced age, lower education and low district HIV prevalence were significantly associated with poor health rating among hypertension patients. The decomposition analysis indicated that 45.5% of urban patients and 36.9% of rural patients reported good self-rated health, representing a statistically significant health gap of 8.6%. Most of the identified health gap can be attributed to endowment effects, with education (62%), district HIV prevalence (26%) and household expenditure (12%) being the most important determinants that explain the health gap.

**Conclusions:**

Urban hypertension patients have better SRH than rural patients in Zambia. Educational interventions, financial protection schemes and strengthening hypertension health services in rural areas can significantly reduce the health gap between the two regions.

## 1. Introduction

### Background

Hypertension is one of the leading causes of premature death (1,2), with over one billion people affected globally. The condition is marked by a chronic elevation of systemic arterial blood pressure above 140/90 mmHg (3,4), which can also lead to other cardiovascular diseases (CVDs) (5,6), such as stroke and ischemic heart disease. Stroke and ischemic heart disease are in turn the top two leading causes of premature death and disability worldwide (7). The burden of hypertension has undergone substantial shifts between and within countries, with poorer countries of low- and middle-income countries (LMICs) bearing a disproportionately high share (8). Moreover, sub-Saharan Africa (SSA) countries have some of the highest hypertension increases and prevalence in the world (9). Within countries, recent evidence shows a steady shift in the burden and distribution of hypertension towards the poorer (8,10,11) and rural communities (12–14). This is in stack contract to over the last decade when the burden of hypertension in most LMICs was mostly concentrated among the affluent and urban population (12,15–17). Poor people are already plagued by infectious disease and hunger. It is therefore crucial to investigate what factors are driving the shift of the burden of hypertension to individuals who are already unequally afflicted by other diseases and poverty. Additionally, while advances in medical care may have generally improved access to care, health systems for noncommunicable diseases (NCDs) are still not adequately prepared to provide the needed interventions for hypertensive and other NCD patients (18–20)

Our main aim is to examine inequalities in overall health status, proxied by self-rated health, among people with hypertension. We do this by first examining how self-rated health among people with hypertension is associated with a rich set of factors. We then examine whether there is a rural‒urban gap in the overall health of people with hypertension and quantify what factors drive this gap. While several studies have looked at inequalities in the burden of hypertension (13,21,22) and access to hypertension treatment (23–27), there are limited or no studies that have documented inequalities in overall health among individuals with hypertension and what could be driving such inequalities – of which access to treatment could be one of the factors.

Understanding systematic differences in health status among people with hypertension would help to highlight that even within people with the same disease condition, some are left behind and suffer more ill health. This may help in setting priorities on what interventions are needed to meet the Sustainable Development Goals (SDGs) on health and reducing inequality (28). There is evidence that hypertension patients who report poor health—based on an indicator called self-rated health (SRH), asking them to rate their health—are at increased risk of cardiovascular events and death (29). Because of its close association with objective measures of health and its simplicity, SRH has been widely used in the literature (30–32). It has been shown that SRH can accurately predict cardiovascular disease, morbidity, mortality, health-seeking behavior, and hospitalization better than diagnosed health (30,33,34) and measures what truly matters—how patients feel (35). Like other measures of health, SRH has multiple determinants, including socioeconomic status (SES), age, sex, location, education, marital status, chronic disorders, and conditions. (36–41). By using SRH, our study contributes to the literature by showing that an easy and cost-effective measure of overall health can be used to quantify inequalities in health within people with the same disease. While clinical measures of severity of disease among people with hypertension are possible, it could be a challenge to use them at a large scale in a population.

It is important to mention that the burden of hypertension and overall health in many regions of sub-Saharan Africa is confounded by a high HIV prevalence. Our study also includes HIV infection in accounting for differences in health status among people with hypertension. The literature suggests that there is a strong association between worsening hypertension and exposure to high HIV/AIDS antiretroviral treatment (ART) (42–48). Our study makes a first attempt at explicitly accounting for this association in examining inequalities in health status among people with hypertension.

Hypertension is a public health problem not only in Zambia but also in the region. In Zambia, approximately 19% of adults aged between 18 and 69 years are hypertensive (49), and the condition is ranked among the top 10 causes of mortality in health institutions for all ages (50). Similar trends have been noted across the African continent and within the region. For instance, 46% of adults aged 25 years and older in Africa are hypertensive (51), with the sub-Saharan African region (SSA) having some of the highest hypertension increases and prevalence rates in the world (9).

What has largely remained unknown, however, are health inequalities among hypertension patients, especially between rural and urban patients. Tracking health inequalities and their determinants is important for identifying the region that may be underserved by health systems and is disproportionately impacted by hypertension (52). This information is likely to result in effective interventions needed to achieve equity-oriented health improvements among patients from various regions (52,53). The current information gap on health inequalities among hypertension patients creates challenges in targeting healthcare resources.

Our study, therefore, provides information that is scantly available within the hypertension literature. Information on rural‒urban health inequalities among hypertension patients is useful for Zambia and other settings with increasing and transitioning hypertension (54). A policy response to manage and control hypertension in these settings requires information, such as rural‒urban inequalities in SRH, and factors explaining the gap in wellness between these patients.

## 2. Methods

### Data and variable description

We utilized a unique household dataset that was specifically designed to collect expenditure and utilization surveys on 11,927 households for health conditions such as hypertension; the Zambia Household Health Expenditure and Utilization Survey (ZHHEUS) (55), for the data on SRH and other demographic and socioeconomic controls. The ZHHEUS is a nationally representative survey conducted in 2015 that used a two-stage stratified cluster sampling design with a sampling frame drawn from the Zambia Census of Population and Housing. The survey aimed to provide information on, among other things, household health-seeking behavior, health service utilization, and expenditure on health. Respondents were asked about any chronic health condition they had had for at least 3 months, including hypertension, diabetes, cancer and HIV/AIDS. These chronic conditions were self-reported as opposed to being diagnosed clinically. Respondents were also asked how they rate their health compared to their agemates, and responses were categorized as “Very Good”, “Good”, “Satisfactory” and “Poor”. In this study, however, these categories were collapsed into either “Good” or “Poor” health.

In addition to the ZHHEUS, we also utilized district HIV prevalence estimates from a previous study (56) to control for district HIV prevalence. The focus on HIV is based on the evidence that HIV and ART have an independent effect on blood pressure and hypertension (57–60). Since there are currently no nationally representative data containing both hypertension and HIV status data at the individual level, we relied on district HIV prevalence data.

Variables of interest for this study included respondents’ hypertension status, demographic characteristics (i.e., age, sex, marital status, region, and education attainment), SRH (categorized into “good” and “poor” health), per capita household expenditure and district HIV prevalence. Note that only individuals with hypertension were included in the study. Table 1 summarizes the variables of interest with their definitions.

### Statistical Analysis

Our study used two statistical models to examine the health gap among people with hypertension and to assess health inequalities between rural and urban patients. First, we used a linear probability model (LPM) to provide a preliminary assessment of the association between independent variables and health. The LPM has been shown to be appropriate for modelling binary dependent variable outcomes if the objective is examining associations, not prediction (61). The advantage of the LPM is that, unlike odds ratios in logistic regression, the coefficients of an LPM are easily interpreted in percentage points without obtaining marginal effects. We then used the Blinder-Oaxaca decomposition to decompose the mean SRH between the rural and urban patients to identify the group with better health and factors contributing to any differences in health between the two groups (62,63). For the purposes of the decomposition, the health variable was also categorized into “Good” and “Poor.” STATA version 15 (56) was used to build the LPM model and conduct the Blinder-Oaxaca decomposition analysis.

The Blinder-Oaxaca decomposition breaks up the factors that contribute to differences in health into three components: the part that is due to group differences in the predictors (known as the endowment), the coefficient component, which accounts for differences in the effects of the predictors, and the part that accounts for the differences in endowments and coefficients that exist simultaneously between urban and rural patients (62,64), known as the interaction component. The endowment effect captures the gap in average health that is explained by inequalities in ownership of factors that are protective of health. On the other hand, the coefficient component accounts for how differences in the effectiveness of factors that are protective of health contribute to the health gap. For example, even if people may have the same level of healthcare, the benefit it confers may be different. This is the coefficient effect. The endowment effect, on the other hand, is having more health because you have access to more healthcare. The interaction is a combination of endowment and coefficient effects. Note that the sum of endowment, coefficient, and interactions should be one hundred percent, so a positive percentage indicates a tendency to increase the health gap, while a negative percentage indicates a tendency to reduce the health gap.

To put it more formally, since the outcome of interest is SRH, i.e., good or poor health $\left(\gamma_{i}\right)$, between urban and rural patients and the analysis is on the differences in the predictors of this health $\left(\chi_{i}\right)$, including the effects of these predictors on health (β), we can think of the Blinder-Oaxaca decomposition process as comprising the following:

(1) $$\gamma_{i}^{u}=\beta^{u}\chi_{i}+\varepsilon_{i}^{u}$$

(2) $$\gamma_{i}^{r}=\beta^{r}\chi_{i}+\varepsilon_{i}^{r}$$

Equations (1) and (2) represent equations linking health to a set of predictors for both urban $\left(\gamma_{i}^{u}\right)$ and rural $\left(\gamma_{i}^{r}\right)$ patients, respectively (65). To explain the gap in health between urban and rural patients, equations (1) and (2) can be rewritten as follows (64,65):

(3) $$\gamma^{u}-\gamma^{r}=\Delta \chi \beta^{r}+\Delta \beta \chi^{r}+\Delta \chi \Delta \beta$$

where $\gamma^{u}-\gamma^{r}$ is the difference in health between rural and urban patients, $\Delta \chi \beta^{r}$ is the endowment component (E), $\Delta \beta \chi^{r}$ is the coefficient component (C) and ΔχΔβ is the interaction term (CE). This can be represented as follows:

(4) $$\gamma^{u}-\gamma^{r}=E+C+CE$$

## RESULTS

### The study sample sociodemographic characteristics

This study analysed data from 2,526 hypertension patients from ZHHEUS. The sample was almost evenly distributed between the urban (50.2%) and rural (49.8%) areas with a median age of 49 years; rural patients were marginally older than the urban patients (median age of 50 years versus 48 years, respectively). Only approximately 19% of urban and 11% of rural patients had attained tertiary education. Table 2 below provides additional sociodemographic characteristics of our sample.

The results of the LPM (Table 3) show that a unit increase in age was associated with a percentage point reduction in the likelihood of reporting good health. In addition, hypertension patients with primary, secondary, and tertiary education were 7 percentage points, 18 percentage points, and 20 percentage points, respectively, more likely to report good health than patients without schooling. Note, however, that the likelihood of reporting good health among those with primary education, compared to those with no schooling, was not statistically significant at the 5% level of significance (p>0.068; 95% CI, −0.005 to 0.143).

Interestingly, hypertension patients residing in districts with high HIV prevalence had a percentage point higher likelihood of reporting a good health rating compared to those in low HIV prevalence districts (p>0.044; 95% CI, 0.0002 – 0.018). There was, however, no significant difference in health ratings between rural and urban patients or between males and females in the adjusted model, despite urban patients being significantly more likely to report good health in the unadjusted model than rural patients. Although lower household expenditure is associated with poorer self-rated health among hypertensive patients, this is not statistically significant in the adjusted model. The study also found that utilizing a healthcare facility was associated with poor self-rated health, albeit not statistically significant.

The LPM clearly shows that age and school attendance are important predictors of self-rated health even after accounting for other factors. The Blinder-Oaxaca decomposition indicated that 45.5% of urban patients and 36.9% of rural patients rated their health as good, resulting in a statistically significant health gap of 8.6% (95% CI, 2.6 to 14.7) [Table 4]. The results show that approximately 104% of the identified health gap can be attributed to endowment effects or how differences in the levels of determinants such as education are distributed.

Specifically, differences in the level of education (62%), district HIV prevalence (26%) and household expenditure (12%) were the most important determinants, within the endowments, that explain the difference in the distribution of SRH between urban and rural patients. This means that if you eliminated differences in these factors, for example, increasing education levels and household expenditure among rural patients, the health gap would decrease significantly. Patients’ age, sex and the level of health facility visits together only contributed approximately a percent towards the health gap between the two regions within the endowments part.

On the other hand, the differences due to the coefficients of the predictor variables explain approximately 73% of the difference in SRH between urban and rural patients. This means that if the variables studied were similarly effective between urban and rural patients, the difference in the way patients feel about their health would decrease by almost 27%, from 8.6% to 6.3%. Age (244%), HIV prevalence (119%), education (107%), household consumption expenditure (3.2%) and service utilization (70.9%) had negative coefficients, suggesting that they were inequality reducing or that their effects on health inequality were in favor of rural patients. On the other hand, the effect of sex (17.5%) worsened health inequalities in favor of urban patients. The constant also had a positive coefficient in favor of urban patients.

The rest of the gap in health between rural and urban patients is explained by the differences in the distribution of determinants in the interactions part (i.e., approximately 77%). Within the interaction part, education (52%), HIV prevalence (24%) and household expenditure (23%) were the main contributors to the health gap and were all inequality reducing. On the other hand, age (0.6%), sex (1.4%) and service utilization (4.5%) had modest contributions to the observed health gap and worsened health inequality in favor of urban patients.

## DISCUSSION

This study examined inequalities in self-rated health between urban and rural hypertension patients and decomposed the determinants contributing to the observed inequalities. Overall, our results show that advanced age and lower education are significantly associated with poor health ratings among hypertension patients. Studies in the United States (US) (36), OECD (Organization for Economic Cooperation and Development) countries (66) and Europe (including Israel) (67) have found similar results. Our study also found that rural hypertension patients generally reported poorer health than their urban counterparts, a finding similar to studies in Iran and Kenya (65,69). The decomposition shows that the level of education, district HIV prevalence and household consumption expenditure explain a substantial part of the health gap between urban and rural patients. Considering that good SRH is an indicator of controlled blood pressure (BP) among hypertensive patients (70), the poor SRH of rural patients is concerning because it may imply lower rates of controlled BP in rural areas.

Our finding that education is a key determinant of health inequality among hypertension patients and that it reduces health inequality in favor of rural patients is consistent with other international studies. For instance, a longitudinal study on the determinants of self-rated health among hypertensive patients in Brazil found an association between lower education and poor self-rated health among patients (81). Similarly, low levels of education were associated with lower quality of life among hypertension patients in Ethiopia (82). This relationship may be attributed to the fact that schooling increases earnings, which in turn facilitate health investment and improve access to health information and service utilization (83,84).

Strategies to reduce health inequalities should therefore focus on investing in and strengthening health education to ensure the adoption of good health habits, such as physical activity, among rural, less educated, and poorer patients. This has been suggested by studies in Brazil and China (85,86). Studies from OECD countries and elsewhere (71,72) have suggested educational interventions as a way to improve health literacy and hypertension management awareness. The role of health literacy in improving the health of hypertension patients cannot be overemphasized. For instance, an integrated review on health literacy and hypertension revealed that health literacy gives patients the ability to make appropriate health decisions (72). Similarly, Huang (74) Shi (75) and Wang (76) found health literacy to be significantly associated with good health in China, while a study among Hispanics in the USA (73) found health literacy to be associated with improved perception in controlling hypertension. Improved health literacy is potentially inequality reducing if targeted towards rural and less educated patients and if accompanied by social welfare programs aimed at assisting target patients to have access to free or low-cost health services (87).

An unexpected finding in our study was that patients residing in high HIV prevalence districts had better health ratings than those from low HIV prevalence districts. This finding suggests that HIV program scale-up, which is predominantly in urban areas, improves not only health systems and access to care but also patients’ SRH. HIV scale-up has been shown to result in improvements in the provision of primary health care and other health services (88,89). For instance, Brugha and colleagues (88) found an upwards trend in service utilization for non-HIV services in the three Zambian districts where HIV services were scaling up. Similarly, the scale-up of HIV services was found to be beneficial to patients with other chronic conditions in South Africa (90). HIV scale-up also increases the number of virally suppressed HIV patients (91) and reduces the onwards transmission of the disease (92). Since HIV prevalence has traditionally been higher in urban districts (93,94), the benefits accruing from improved health systems, due to HIV scale up, are likely to benefit urban patients more (95,96), thereby positively affecting their health perception and SRH. Our study highlights synergies between HIV and hypertension health services. Therefore, there is a need to identify health system gaps in rural areas in an effort to improve overall health outcomes among rural hypertension patients (97).

Another important finding is that service utilization is inequality reducing, in favor of rural patients. Currently, health service utilization is not only concentrated among urban patients but also more effective in urban areas, thereby partially explaining the existing health gap between the two regions. The access gaps in rural areas may be attributed to weak healthcare systems, distance to facilities, staff shortages, etc., as revealed by other studies in Zambia (21) and in the region (98,99). Reducing health inequalities between urban and rural hypertensives entails addressing these system inadequacies and improving service utilization.

Health inequalities are likely to persist as long as tertiary and specialized health services remain in urban areas, thereby benefiting urban patients more (100). Such inequitable distribution of services has resulted in better control of hypertension for urban patients compared to their rural counterparts in Ghana (101) Colombia (102) and China (103). In addition, inequalities in the type and quality of hypertension services between rural and urban areas are a potential driver of downstream inequities, such as differences in health outcomes and health perceptions.

Therefore, there is a need for strategies aimed at ensuring that rural hypertension patients have control of their hypertension condition due to the positive association between controlled hypertension and self-rated health (104). Jongen and others found community-based hypertension awareness activities to be effective in managing and controlling hypertension among the rural poor in South Africa (105). Other studies on hypertension and self-rated health in Brazil (81) and China (104) have also documented the effectiveness of social integration and community support groups in improving patients’ self-rated health. These ‘community health systems’ should, therefore, be nurtured and encouraged among rural hypertension patients in an effort to improve their health. In addition, rural patients with hypertension and other NCDs should be targeted for inclusion in financial protection schemes such as the National Health Insurance (NIH) scheme and financially empowered through social cash transfers to reduce out-of-pocket health expenditures (106,107). This financial empowerment is necessary for timely access to care and promotion of choices for healthy lifestyles among rural patients of most LMICs (105,108).

## LIMITATIONS

This study utilized cross-sectional observational data to examine health inequalities between rural and urban hypertension patients. The hypertension status of the respondents was self-reported and not clinically confirmed, which may result in reporting bias. This also means that those who were hypertensive but did not know their status were excluded. In addition, caution is required when making conclusions on the observed association between SRH and HIV prevalence because health status is at the individual level, while HIV prevalence is at the population level. However, the association between these variables provides useful insights into the interaction between patients’ perceived health and HIV dynamics in their communities. This relationship may need further investigation by future studies using appropriate data. Furthermore, attributing a causal relation between health and other investigated variables using cross-sectional data may be problematic and should be done with caution (109,110).

## CONCLUSION

Our study revealed that health inequalities exist among hypertension patients in Zambia, with urban patients having better SRH than rural patients. Patients with poor health ratings were older, less educated and had lower socioeconomic status. Policy initiatives to improve health and reduce health inequalities across geographic regions should target patients in rural areas, with intentions of improving their health literacy and access to hypertension services.

Educational interventions and financial protection schemes for rural patients can empower them to make appropriate and timely decisions on accessing healthcare services and managing their condition. Some LMICs have introduced financial protection policies, such as the NHI scheme in Zambia and social cash transfer programs, and these can be systematically targeted towards rural hypertension patients to improve their health-seeking behavior and health.

There are indications from our study that strengthening hypertension services and other related services can positively influence patients’ health perception. Hypertension patients from high HIV prevalence districts, where hypertension services are likely to be performing better due to improved HIV services, have better health ratings than patients from lower HIV prevalence districts. This finding reveals the existence of important synergies that can be taken advantage of to improve the delivery of hypertension services. Strengthening community health services can be an important first step to strengthen rural health systems. A community strategy similar to the one used by the Better Health Outcomes through Mentoring and Assessment (BHOMA) approach (111) can help improve the delivery of hypertension services and the health of patients in rural areas.

The decomposition analysis has provided valuable information on the specific factors contributing to health inequality between rural and urban hypertension patients in Zambia. This information is important for policies aimed at improving patients’ health outcomes. The use of such methodology is encouraged if resource-limited settings have to put the resources where the needs are.

## Figures and Tables

**Table 1:** List of variables

Variable	Variable definition	Coding
Age	Age of respondent at last birthday in years	Continuous numeric values
Sex	Sex of respondent	Female (0) Male (1)
School Attendance	Respondent’s highest level of formal education	No school (0) Primary (1) Secondary (2) Tertiary (3)
District HIV prevalence	Prevalence of HIV at district level	Continuous numeric values
Marital status	Current marital status of respondent	Never married (1) Married (2); Separated/divorced/widowed (3)
Region	Respondents’ residence	Rural (0) Urban (1)
Self-Rated Health	How the respondent rates his/her health in comparison to others of his/her age	Poor (0) Good (1)
Per-capita household expenditure	Average expenditure on goods and services by each household member	Continuous numeric values
Service utilization	Whether any household member was sick and visited a health care facility in the 4 weeks preceding the survey	No (0) No (1)

**Table 2:** Sociodemographic characteristics of the study sample

	Urban (N=1267)	Rural (N=1259)	Total (N= 2,526)

	Freq. (%)	Freq. (%)	Freq. (%)

**Age**			
Median/(Mean)	48/(48.3)	50/(48.8)	49/(48.6)
**Sex**			
Male	382 (30.1)	345 (27.4)	727 (28.8)
Female	885 (69.9)	914 (72.6)	1,799 (71.2)
**Marital status**			
Never married	104 (8.2)	90 (7.2)	194 (7.8)
Married	777 (61.3)	823 (65.4)	1,600 (63.3)
Separated/divorced	386 (30.5)	346 (27.5)	732 (28.9)
**Self-Rated Health**			
Poor	515 (40.7)	673 (53.5)	1,188 (47.0)
Good	752 (59.4)	586 (46.5)	1,338 (52.9)

**District HIV Prevalence**			
Median/(Mean)	14.3/(13.7)	10.5/(11)	12.1/(12.4)
**School Attendance**			
No school	139 (10.9)	272 (21.6)	411 (16.3)
Primary	391 (30.9)	693 (55)	1,084 (42.9)
Secondary	502 (39.6)	254 (20.2)	756 (29.9)
Tertiary	235 (18.6)	40 (3.2)	275 (10.9)
**Service Utilization**			
Yes	303 (23.9)	390 (30.9)	693 (27.4)
No	145 (11.4)	220 (17.5)	365 (14.5
Non response	819 (64.6)	649 (51.6)	1,468 (58.1)

**Table 3:** Association between health and independent variables in a Linear Probability Model (LPM)

N=2,526	Crude Coef. (95% CI)	p value	Adjusted Coef. (95% CI)	p value

**Age**	−0.0086 (−0.0097, 0.0074)	<0.001	−0.0068 (−0.0088, −0.0049)	<0.001
**Sex**				
Male	***Ref***			
Female	−0.027 (−0.072, 0.018)	0.242	−0.0145 (−0.0867, 0.0576)	0.693
School Attendance				
No school	***Ref***			
Primary	0.106 (0.049, 0.162)	<0.001	0.069 (−0.0050, 0.1435)	0.068
Secondary	0.256 (0.193, 0.319)	<0.001	0.176 (0.078, 0.274)	<0.001
Tertiary	0.303 (0.231, 0.376)	<0.001	0.202 (0.064, 0.340)	0.004
**District HIV Prevalence**	0.013 (0.0075, 0.0182)	<0.001	0.0089 (0.00024, 0.0175)	0.044
**Region**				
Rural	***Ref***			
Urban	0.141 (0.095, 0.188)	<0.001	0.020 (−0.055, 0.095)	0.598
**Per-capita Household consumption Expenditure**	3.73 (−4.33, 0.000012)	0.364	−6.52 (−0.000014, 1.18)	0.097
**Service Utilization**				
No	***Ref***			
Yes	−0.004 (−0.066, 0.058)	0.902	−0.021 (−0.0832, 0.0413)	0.508

**Table 4:** Blinder-Oaxaca decomposition of health status among individuals with hypertension in Zambia

	Coeff	Percentage contribution

Mean health for urban patients	45.5	
Mean health for rural patients	36.9	
**Difference in health**	8.6	
*Due to endowments*	8.9	103.5
*Due to coefficients*	6.3	73.2
*Due to interactions*	−6.6	−76.7

**Endowments**		
*Age*	0.08	0.9
*Education*	5.5	61.8
*HIV prevalence*	2.3	25.8
*Sex*	−0.04	−0.4
*Per-capita exp*	1.1	12.4
*Service utilization*	0.05	0.6

**Coefficients**		
*Age*	−15.4	244
*Education*	−6.6	107
*HIV prevalence*	−7.5	119
*Sex*	1.1	17.5
*Per-capita exp*	−0.2	3.2
*Service utilization*	−4.4	70.9
*Constant*	25.5	405

**Interaction**		
*Age*	0.04	0.6
*Education*	−3.4	−51.5
*HIV prevalence*	−1.6	24.2
*Sex*	0.09	1.4
*Per-capita exp*	−1.5	−22.7
*Service utilization*	−0.3	−4.5

## Data Availability

The data and materials used for this study are available from the corresponding author on request.

## LIST OF ABBREVIATIONS

LMICs: Low- and middle-income countries
SSA: Sub-Saharan Africa
NCDs: Noncommunicable diseases
SDGs: Sustainable development goals
SRH: Self-rated health
ART: Antiretroviral therapy
ZHHEUS: Zambia Household Health Expenditure and Utilization Survey
LPM: Linear probability model
OECD: Organization for Economic Cooperation and Development
NHI: National Health Insurance
